# Exocyst subunit SEC3A marks the germination site and is essential for pollen germination in *Arabidopsis thaliana*

**DOI:** 10.1038/srep40279

**Published:** 2017-01-11

**Authors:** Yan Li, Xiaoyun Tan, Mengru Wang, Bingxuan Li, Yanxue Zhao, Chengyun Wu, Qingchen Rui, Junxia Wang, Zhongyuan Liu, Yiqun Bao

**Affiliations:** 1 College of Life Sciences, Nanjing Agricultural University, Nanjing 210095, People’s Republic of China

## Abstract

Arabidopsis exocyst subunit SEC3A has been reported to participate in embryo development. Here we report that *SEC3A* is involved during pollen germination. A T-DNA insertion in *SEC3A* leads to an absolute, male-specific transmission defect that can be complemented by the expression of *SEC3A* coding sequence from the *LAT52* promoter or *SEC3A* genomic DNA. No obvious abnormalities in the microgametogenesis are observed in the *sec3a/SEC3A* mutant, however, *in vitro* and *in vivo* pollen germination are defective. Further studies reveal that the callose, pectin, and cellulose are apparently not deposited at the germination site during pollen germination. *SEC3A* is expressed ubiquitously, including in pollen grains and pollen tubes. Notably, SEC3A-GFP fusion proteins are specifically recruited to the future pollen germination site. This particular localization pattern is independent of phosphatidylinositol 4,5-bisphosphate (PI-4,5P_2_), although SEC3-HIS fusion proteins are able to bind to several phosphoinositols *in vitro*. These results suggest that *SEC3A* plays an important role in the establishment of the polar site for pollen germination.

Pollen germination is a very important event among a series of pollination processes by which pollen tube delivers the sperm cells into the ovule to complete fertilization. In Arabidopsis, once a desiccated, pollen grain contacts a papilla cell on the stigmatic surface, it became hydrated within a short period of time. The presence of a Ca^2+^ gradient beneath the potential germination site^1^,^2^, the reorganization of F-actin cytoskeleton^3^,^4^, and the massive deposition of callose, pectin, and cellulose at the germination plaque^5^,^6^,^7^ are key events taking place before tube emergence. Until now, it is not yet clear how the the germination site is established.

In order to satisfy pollen germination and the rapid pollen tube tip growth, cell wall material, proteins and other membrane components, are transported to the growing tip of the pollen tube via the vesicle trafficking system^8^,^9^. Compared to intensive researches on pollen tube growth, limited number of players functioning from the onset of pollen germination have been identified. These included proteins involved in cell wall material synthesis and modification. For example, *BUP* encodes a novel Golgi-located glycosyltransferase, the *bup* is affected during pollen germination and pollen tube growth by affecting pectin synthesis or delivery^7^. Pollen grains from homozygous plants mutated in *PECTIN METHYLESTERASE48* gene developed multiple germinating sites due to the presence of more abundant highly methylesterified pectin in the intine wall^10^. A T-DNA insertion line mutated in the *Callose Synthase 9* gene produced pollen grains able to *in situ* precociously germinate inside the anther^6^, and mutations in *CSLD1* and *CSLD4* caused a significant reduction in cellulose deposition and an alteration of the cell wall organization leading to defective pollen germination and tube growth^11^. These data suggested that tight regulation of cell wall synthesis and modification is crucial for the germination site establishment and pollen tube emergence. Additionally, components involved in vesicle trafficking, an intimately related process for cell wall material delivery, have also been implicated during pollen germination^12^. For example, a mutation in *AtSYT2*, a homolog of mammalian Synaptotagmins implicated in regulating membrane fusion during exo/endocytosis, affected pollen germination and pollen tube elongation^13^,^14^,^15^. Pollen-specific GNL2 was shown to be essential for pollen germination and pollen tube tip growth based on its necessary role in polar recycling. GNL2 is localized to the germination site and pollen tube tip, and absolutely no pollen germination was observed in *gnl2* mutants^16^,^17^. Furthermore, proteins involved in intracellular signaling^18^, and dynamic actin regulation^3^,^4^ have also been shown to play a role during pollen germination.

Vesicle tethering is required after vesicle delivery but preceding the SNARE-mediated docking/fusion steps at the target membrane^19^. The exocyst, composed of SEC3, SEC5, SEC6, SEC8, SEC10, SEC15, EXO70, and EXO84 subunits, is an evolutionally conserved octameric protein complex that tethers secretory vesicles to specific domains of the plasma membrane^20^. Spatial regulation of membrane trafficking by the exocyst complex is fundamental to epithelial cell polarization, neuronal synaptogenesis and the polar growth of budding yeast^21^, which suggested a role of exocyst in polar exocytosis. Several Arabidopsis exocyst subunits have been implicated in biological processes that rely on regulated vesicle trafficking. For example, mutations in *SEC5, SEC6, SEC8*, and *SEC15A* dramatically reduced pollen germination and pollen tube growth which led to a male-specific transmission defect^22^,^23^. Mutations in *SEC8* and *EXO70A1* locus reduced pectin deposition in the seed coat^24^. *exo70a1, exo84b*, and pollen rescued *sec6* mutants (*PRsec6*) display cytokinesis defects^25^,^26^. In maize *roothairless1* where *SEC3* was mutated, root hairs could not elongate properly^27^.

Previous investigations of the *sec3a* mutation (SALK_145185) indicated that the deletion of *SEC3A* gene resulted in embryo-lethality. The authors showed that in the interphase cells, SEC3A-GFP is present in the cytosol and at the plasma membrane where it accumulates as immobile punctate structures over the cell surface of the root hairs and the root epidermal cells, furthermore its recruitment to the plasma membrane is not mediated by the conventional secretory pathway^28^.

In this report, with another T-DNA insertion mutant of the *SEC3A* gene (GK_652H12), we provided genetic, molecular, and cellular evidence that SEC3A is crucial for male gametophytic transmission. The mutation in *SEC3A* gene led to the lack of pollen germination along with perturbations in the deposition of cell wall material. SEC3A-GFP fusion protein was found to accumulate at the future site of pollen germination, which is independent on PI-4,5P_2_ content. These results suggest that the polar localization of SEC3A at the bulge of the germinating pollen grain is essential for pollen germination.

## Results

### The expression pattern of *SEC3A* and *SEC3B* genes

**Figure 1:**
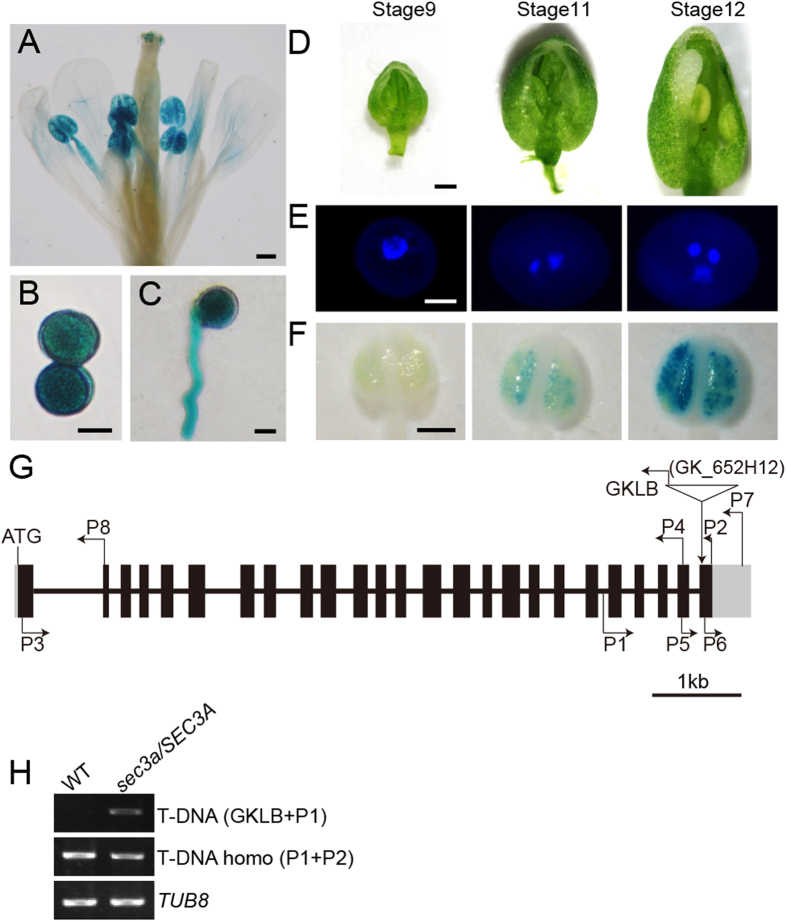
*SEC3A* expression pattern in pollen and the genotyping of *sec3a/SEC3A* mutant. (**A**) The flower, (**B**) Mature pollen grains, and (**C**) Pollen tube showed strong *SEC3A* expression. (**D**) Flowers at stage 9, 11, 12 were used for anther dissection. (**E**) DAPI staining showed that pollen from stage 9, 11, 12 flowers was at unicellular, bicellular, and the tricellular stages, respectively. (**F**) GUS staining of anthers from flowers of (**D**). (**G**) A schematic representation of *SEC3A* transcript and the site of T-DNA insertion. The T-DNA insert is located in the last Exon. The black boxes represent exons, lines in between represent introns. GKLB, P1, P2 are the primers used in the PCR assays. (**H**) Genotyping of *sec3a/SEC3A* by PCR analysis. *TUB8* was used as an internal control. Bars = 0.2 mm for (**A**,**F**), 10 μm for (**B**,**C**), 0.4 mm for (**D**), and 5 μm for (**E**).

The time and location of gene expression often implies its function. To investigate the expression pattern of *SEC3A* in detail, a 1024 bp promoter region upstream from the ATG start codon was fused with a *GUS* reporter gene and introduced into wild type Arabidopsis plants. Fifteen independent *pSEC3A:GUS* transgenic plants were obtained and six of them were chosen randomly and examined further. Strong GUS activities were detected in flowers (Fig. 1A), mature pollen (Fig. 1B) and pollen tubes (Fig. 1C). Notably, *SEC3A* started its expression only in pollen from the bicellular stage (Fig. 1D,F), which corresponded to the stage 11 flower onwards^29^ (Fig. 1E,F). In addition, *SEC3A* expression was detected in seedlings, root columella cells, vascular bundles of root maturation zone, and embryos (Supplementary Fig. S1), suggesting a role of SEC3A in sporophytic development as well.

In Arabidopsis, *SEC3B* shares 96.6% sequence identity to *SEC3A* in the coding region, and the two genes are arranged in tandem. It is therefore very important to explore the expression pattern of *SEC3B* to predict whether they are functionally redundant in a particular tissue. In four *pSEC3B:GUS* lines obtained, the GUS staining of *SEC3B* looked much weaker than that of *SEC3A* which is consistent with the RT-PCR results generated with gene-specific primers (Supplementary Fig. S2, Supplementary Table S1). *SEC3B* expression was not detected in the pollen and pollen tube, although it was noticed in tissues such as the cotyledon, the root vascular bundles, and the mature leaves (Supplementary Fig. S3). Furthermore, homozygotes obtained from the progeny of two *sec3b/SEC3B* mutants (SALK_071060, SALK_124458) showed no observable phenotype. These results indicate that *SEC3A* is the major *SEC3* paralog in pollen.

### A mutation in *SEC3A* gene causes male sterility

*SEC3A* gene contains 25 exons and 24 introns. One T-DNA line of the *SEC3A* gene (*sec3a/SEC3A*, GK_652H12) was obtained where the insertion was in the last exon (Fig. 1G). PCR-based genotyping revealed that no homozygous *sec3a* mutant plants could be identified (n > 180) (Fig. 1H). The progeny from the self-pollinated *sec3a/SEC3A* plants segregated in a ratio of roughly 1:1 (n = 243) instead of the expected 3:1 (Table 1), indicating a disorder in gametophytic transmission rather than a zygotic lethality of the mutation. To investigate if the mutation in *SEC3A* affected male or female gametophytic development, the *sec3a/SEC3A* plants were used as male or female donors to cross with the wild type. When the *sec3a/SEC3A* plants were crossed as female parents, approximately 49% (n = 271) of the resulting F1 progenies were heterozygotes as expected for normal transmission. In contrast, when pollen grains from the *sec3a/SEC3A* were pollinated to wild type plants, none (n = 224) of the F1 seedlings were heterozygous plants (Table 1). These results indicated that genetic transmission of *sec3a* mutation through the male was abolished in the mutant, while female gametophytic transmission was normal.

### The *sec3a* mutant is defective during pollen germination

**Figure 2:**
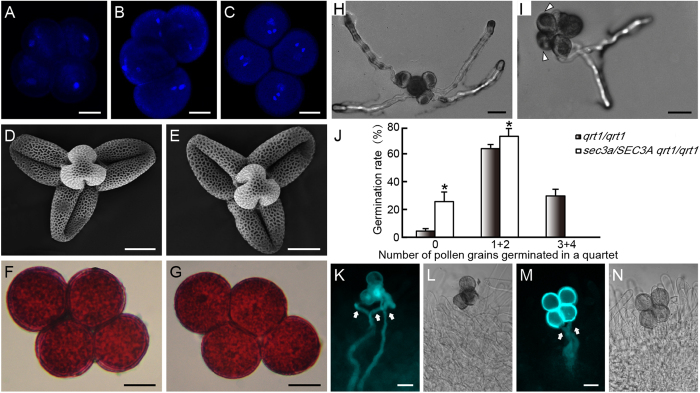
*sec3a* pollen is defective during pollen germination. Quartets from *sec3a/SEC3A qrt1/qrt1* plants was stained with DAPI at the unicellular (**A**), bicellular (**B**) and tricellular (**C**) stages. Scanning electron microscopy (SEM) analysis of *qrt1/qrt1* (**D**) and *sec3a/SEC3A qrt1/qrt1* quartets (**E**). Alexander staining of quartets from *qrt1/qrt1* (**F**) and *sec3a/SEC3A qrt1/qrt1* (**G**). No abnormality was observed for *sec3a/SEC3A qrt1/qrt1* quartets in above assays. *In vitro* germination of quartets of *qrt1/qrt1* (**H**) and *sec3a/SEC3A qrt1/qrt1* (**I**) plants on solid germination medium. (**J**) Statistic analysis of *qrt1/qrt1* and *sec3a/SEC3A qrt1/qrt1* quartets showing 0, 1 + 2, 3 + 4 pollen tube(s) 6 h after germination. Values represent the means ± SD. ^*^Means *P* < 0.05 by Student’s *t* test (n = 600 pollen grains for each genotype). *In vivo* germination of pollen grains from *qrt1/qrt1* (**K**,**L**) and *sec3a/SEC3A qrt1/qrt1* (**M**,**N**) quartets. (**L**,**N**) are corresponding bright-field images of (**K**,**M**), respectively. Arrowheads in (**I**) indicated non-germinated pollen grains. Arrows in (**K**,**M**) indicated pollen tubes. Bars = 10 μm for (**A**–**G**), 20 μm for (**H**,**I**,**K**,**M**).

Male gametophytic lethal might be due to two possible defects. One is that haploid cells inherited the mutant allele after meiosis do not develop into mature pollen, or they developed normally, but are defective during pollen germination and/or tube growth, or later stages of double fertilization. To address these two possibilities, *sec3a* was introgressed into the *quartet 1 (qrt1*) mutant background where four microspores from a microsporocyte fail to separate after meiosis, but their functions are virtually unaffected^30^,^31^. Within a quartet, two microspores are mutant (*sec3a*) and the other two are wild type (*SEC3A*). Pollen grain development was examined using the quartets from *sec3a/SEC3A qrt1/qrt1* plants. No difference in nuclear composition was observed in quartets at the unicellular, bicellular and tricellular stages using DAPI staining (Fig. 2A–C). In addition, *sec3a* pollen grains were morphologically comparable to wild type under the scanning electron microscope (Fig. 2D,E), and they are viable as revealed by the Alexander staining (Fig. 2F,G). Together, these results indicated that the mutation in *SEC3A* did not affect pollen development.

Pollen germination *in vitro* and *in vivo* were carried out with quartets of *sec3a/SEC3A qrt1/qrt1* (Fig. 2). Due to the 2:2 ratio of the wild type versus mutant within a quartet from a heterozygote, germination of three or four pollen grains in the quartet requires the germination of one or both mutant pollen grains. Therefore, a relative low percentage of quartets with three or four grains germinated would indicate a pollen germination defect. When cultured on a solid medium, approximately 31.7% (n = 600) of quartets from *qrt1/qrt1* plants had three or four pollen grains germinated (Fig. 2H,J), while no quartet from *sec3a/SEC3A qrt1/qrt1* (n = 600) plants could do the same (Fig. 2I,J). *In vivo* pollination assay was carried out using male-sterile plants (*ms1*) as pollen recipient to avoid the potentially complicating effects of stigma maturity and emasculation stresses^32^. While approximately 47% (n = 108) of *qrt1/qrt1* quartet could germinate three or four pollen tubes into the pistil as revealed by aniline blue staining (Fig. 2K,L), none of the *sec3a/SEC3A qrt1/qrt1* (n = 60) quartets was able to do that (Fig. 2M,N). These results suggested that *sec3a* mutation significantly inhibited pollen germination *in vitro* and *in vivo*.

### Phenotype of *sec3a/SEC3A* is rescued by *pLAT52:SEC3A, pLAT52:SEC3A-GFP* and *gSEC3A* transgenes, respectively

**Figure 3:**
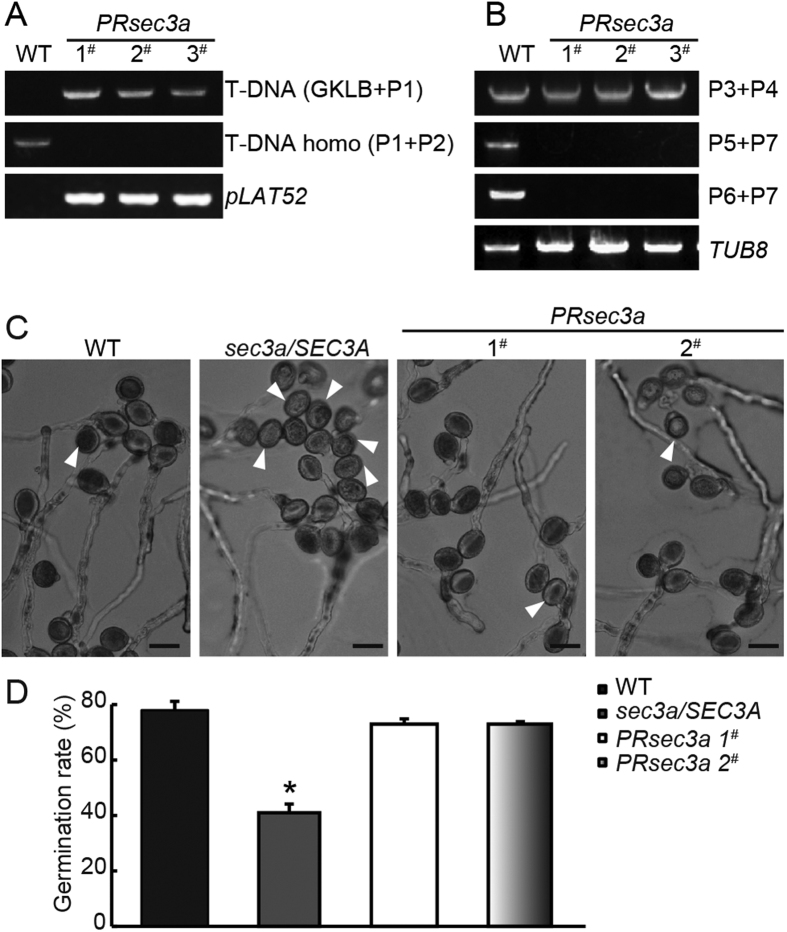
Complementation of *sec3a* mutants with *pLAT52:SEC3A* transgene. (**A**) Genotyping of *PRsec3a*. (**B**) RNAs were extracted from true leaves, and P3 + P4, P5 + P7 or P6 + P7 primer pairs were used to analyze different *SEC3A* transcripts. (**C**) *In vitro* germination of pollen grains from wild type, *sec3a/SEC3A*, and *PRsec3a* plants after 6 h on the solid medium. Arrowheads indicated ungerminated pollen. (**D**) Statistic analysis showed that *pLAT52:SEC3A* transgene restored *in vitro* germination rate of *sec3a/SEC3A* pollen to normal. Values represent the means ± SD. ^*^Means *P* < 0.05 by Student’s *t* test. Bars = 20 μm.

To demonstrate that the mutant phenotype is caused by the disruption of *SEC3A* gene, *sec3a/SEC3A* plants were transformed with *SEC3A* coding sequence driven by the pollen-specific *LAT52* promoter^33^ in a vector that confers resistance to hygromycin. Transgenic plants hemizygous for *pLAT52:SEC3A* (20 lines) and *pLAT52:SEC3A-GFP* (12 lines) loci in *sec3a/SEC3A* mutant were obtained, and several lines of each genotype were chosen randomly for further characterization. The male transmission defects were complemented, with *sec3a/sec3a, sec3a/SEC3A* and wild type progeny appearing at the expected Mendelian ratio (Table 2). Moreover, *sec3a* homozygotes, named as *PRsec3a* (Pollen-Rescued *sec3a: sec3a/sec3a pLAT52:SEC3A/pLAT52:SEC3A*), were identified using PCR-based genotyping with progeny lines in which all seedlings showed hygromycin resistant (*pLAT52:SEC3A* transgene selection marker) (Fig. 3A). RT-PCR analysis of *PRsec3a* mutants indicated that the transcripts spanning (P5 + P7) or behind (P6 + P7) the T-DNA insertion sites were not expressed, while the transcript (P3 + P4) before the insertion was detected (Figs 1A and 3B). However, it should be unstable due to the lack of *Poly(A)* tail. Similarly, *PRsec3a-GFP* lines (*sec3a/sec3a pLAT52:SEC3A-GFP/pLAT52:SEC3A-GFP*) were obtained (Table 2, Supplementary Fig. S4). Furthermore, twelve *sec3a/SEC3A* lines bearing hemizygous *SEC3A* genomic DNA were generated (Supplementary Fig. S4). In two randomly selected lines, the male transmission efficiency increased to approximately 2:1, indicating a complete complementation (Supplementary Table S2).

*In vitro* pollen germination of two randomly selected *PRsec3a* lines were evaluated on the solid media. Under the same conditions, 78% of wild type pollen grains (n = 672) and 41% (n = 638) of that from *sec3a/SEC3A* plants were able to germinate (Fig. 3C,D). Remarkably, in *PRsec3a* mutant, the ratio was restored to that of the wild type (73%, n = 683) (Fig. 3C,D). These date demonstrated that the male gametophytic defects were indeed due to T-DNA insertion in the *SEC3A* gene.

### SEC3A-GFP decorates the pollen germination site, and the localization is independent on PI-4, 5P_2_

**Figure 4:**
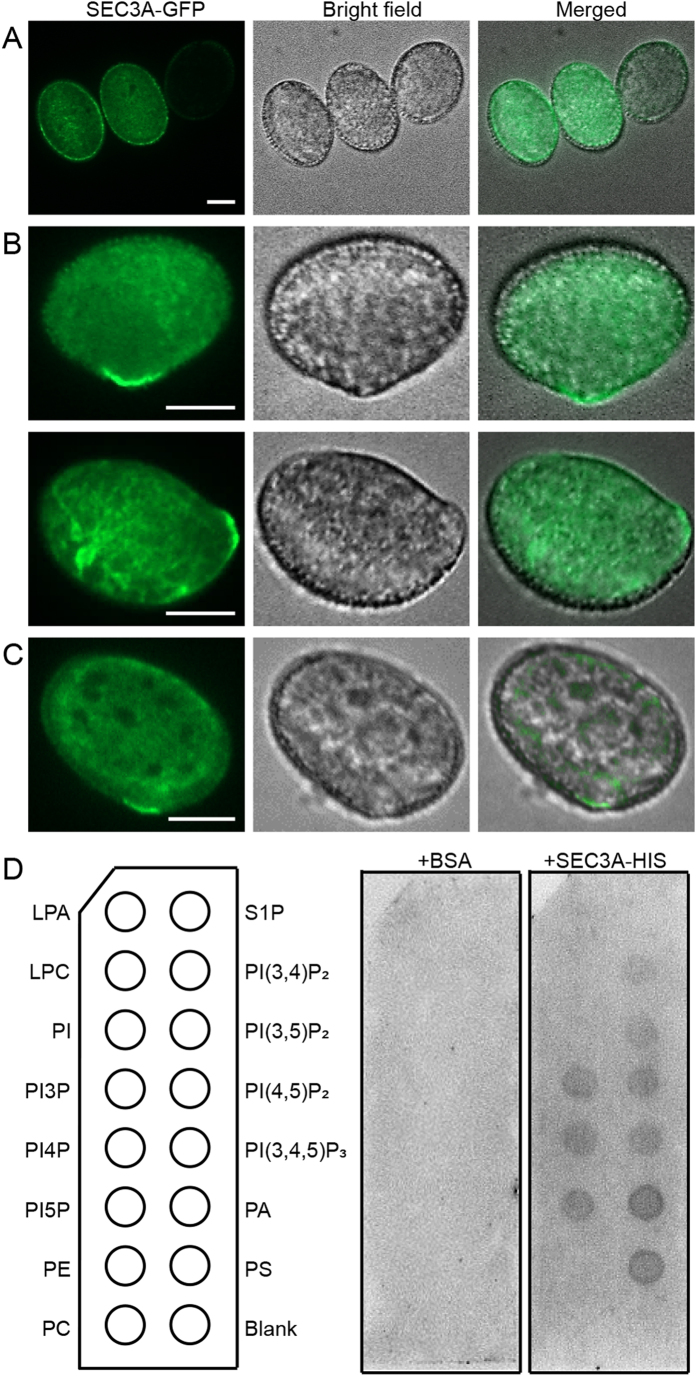
SEC3A proteins marks the pollen germination site, and this localization is independent on PI4, 5-P_2_. *pLAT52:SEC3A-GFP* was transformed into wild type (Col-0) (**A,B**), and *pip5k4* mutant plants (**C**), respectively, and pollen grains were collected and germinated in liquid medium. (**A**) SEC3A-GFP localization before pollen germination. (**B**) SEC3A-GFP localization during pollen germination. (**C**) SEC3A-GFP localization during pollen germination in *pip5k4* mutant background. (**D**) SEC3A protein lipid overlay binding assay. Bars = 10 μm.

To analyze the role of SEC3A more precisely, the dynamic localization of SEC3A in the germinating pollen grains were explored. Our studies showed that the male transmission was restored to normal in *sec3a/SEC3A* mutant with hemizygous *pLAT52:SEC3A-GFP* (Table 2), and *PRsec3a-GFP* lines could be identified (Supplementary Fig. S4). These results indicated that the SEC3A-GFP protein is functional in pollen, and therefore should display its correct subcellular localization. In *pLAT52:SEC3A-GFP* transgenic plants, SEC3A proteins appeared from the binucleated pollen stage onwards (Supplementary Fig. S5), consistent with the GUS analysis results (Fig. 1). In mature pollen prior to activation, SEC3A-GFP was dispersed in the cytoplasm (Fig. 4A). Interestingly, after being placed in liquid germination medium for 30 min, SEC3A-GFP was shift to the cortex of the future germination site before any visible change of pollen morphology was noticed (Fig. 4B, Supplementary Video. S1), and the signals oscillated at this position throughout the tube emerging process (Supplementary Videos S1 and S2).

In yeast, Sec3p directly interacted with PI-4,5P_2_ and marked the exocytic site at the bud^34^. To test whether SEC3A could bind to PIPs directly *in vitro*, SEC3A was fused to HIS and expressed in *E. Coli*. Purified proteins were used in PIPs strip overlay assays. As shown in Fig. 4D, SEC3A bound to several membrane phosphoinositides including PI-4,5P_2_, while the control with BSA alone could not (Fig. 4D). In plants, PI-4,5P_2_ could be generated through the phosphorylation of phosphatidylinositol-4-phosphate (PI4P) by phosphatidylinositol-4-phosphate 5-kinases (PIP5K)^35^,^36^. In *pip5k4* mutant, the production of PI-4,5P_2_ and the membrane recycling was decreased in pollen tubes^37^,^38^. *pLAT52:SEC3A-GFP* was introduced into the *pip5k4* homozygous mutant (SALK_001138, Supplementary Fig. S6), and its localization was examined. Interestingly, SEC3A proteins still accumulated at the bulge of the germinating *pip5k4* pollen grain with bright fluorescent labeling (Fig. 4C), just as in the wild type (Fig. 4B), indicating that the localization of SEC3A in pollen was not dependent on PI-4,5P_2_.

### Polar accumulation of cell wall material was not observed in the germinating *sec3a* pollen grains

**Figure 5:**
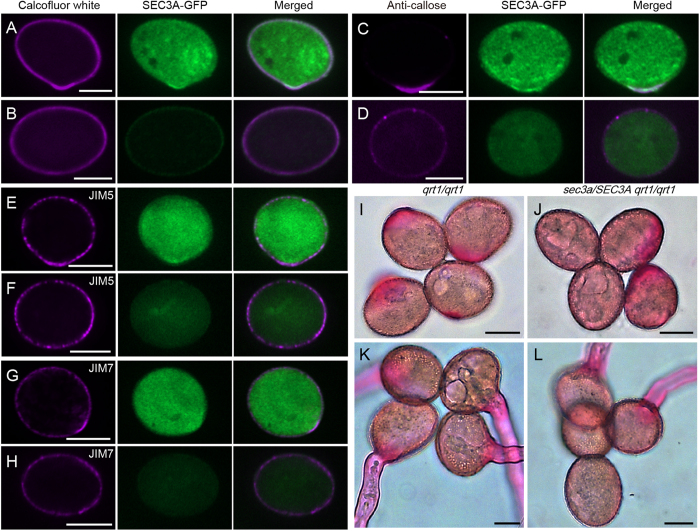
Accumulation of Cell wall materials are not observed in *sec3a* germinating pollen. (**A**,**B**) Calcofluor white staining. (**C,D**) Pollen grains labeled with anti-callose monoclonal antibody. (**E,F**) Pollen grains labeled with JIM5 monoclonal antibody. (**G,H**) Pollen grains labeled with JIM7 monoclonal antibody. *sec3a pLAT52:SEC3A-GFP* (**A**,**C,E,G**) and *sec3a* (**B**,**D,F,H**) pollen were used for the assays. (**I**–**L**) Ruthenium red staining of tetrads from *qrt1/qrt1* (**I**,**K**) and *sec3a/SEC3A qrt1/qrt1* (**J**,**L**) plants. Bars = 10 μm.

Pollen germination started with hydration, which might act as a triggering signal. Following hydration, plaque formation starts and is completed within 1 h, by then the pollen tube has emerged^39^. Polar deposition of callose, cellulose and pectin is observed in the incipient bulge of germinating pollen grains^5^,^7^,^40^. The phenotypes of *sec3* mutant and the localization pattern of SEC3A prompted us to look into the cell wall deposition at the germination site. Pollen grains from *sec3a* mutant hemizygous for *pLAT52:SEC3A-GFP* were used since mutant pollen (*sec3a*) can easily distinguished from the complemented one (*sec3a pLAT52:SEC3A-GFP*) by the absence or presence of the GFP signal. Calcofluor white which labels cellulose and callose^41^, appeared at the germination site in the complemented pollen grain, colocalized with the SEC3A-GFP signals (Fig. 5A). Similarly, antibodies against callose (anti-callose, Fig. 5C), low methylestified pectins (JIM 5, Fig. 5E), and high methylestified pectins (JIM7, Fig. 5G) labeled the germination plaques in the complemented pollen grains, respectively. In contrast, mutant pollen exhibited an even staining of the cell wall with no sign of germination (Fig. 5B,D,F,H). In addition, *qrt1/qrt1* and *sec3a/SEC3A qrt1/qrt1* quartets were stained with ruthenium red, which labels a broad range of methylesterified pectins. Ruthenium red strongly labeled the germination plaques of all four pollen grains of *qrt1/qrt1* (Fig. 5I) while only two pollen grains of *sec3a/SEC3A qrt1/qrt1* quartet could be labeled (Fig. 5J). Even after 6 h of germination when a quartet from *qrt1/qrt1* plant produced four pollen tubes (Fig. 5K), *sec3a/SEC3A qrt1/qrt1* quartet still had two non-germinated pollen grains that show no sign of polar pectin deposition (Fig. 5L). Thus pectin distribution revealed by ruthenium red is consistent with the JIM5 and JIM7 staining (Fig. 5E,F,G,H). Together, these data demonstrated that polar accumulation of several types of cell wall materials was not observed in the germinating *sec3a* pollen.

## Discussion

In this report, we demonstrated that a mutation in *SEC3A* led to the failure of pollen germination with no polar accumulation of cell wall materials. *SEC3A* is expressed in pollen and pollen tube, and SEC3A marked the pollen germination site. Thus, SEC3A is a key player during pollen germination, and its recruitment to the germination site is not dependent on PI4,5-P_2_.

Mutations in distinct exocyst subunit caused polar growth defects in processes such as root hair elongation, hypocotyl elongation, and pollen tube growth^22^,^23^,^27^,^42^. In this study, we found that in *sec3a* mutant (GK_652H12), germination of the pollen grains was hindered, resulting in an absolute male-specific transmission defect which could be rescued by *pLAT52:SEC3A, pLAT52:SEC3A-GFP* or *genomic SEC3A* transgene (Figs 2 and 3, Table 2, Supplementary Fig. S4, Supplementary Table S2). The *sec3a* pollen grains developed normally, however, no pollen germination was observed in *in vitro* and *in vivo* germination assays (Fig. 2), let alone pollen tube growth. In a recent paper^43^, the authors showed that among 1769 sec3a heterozygous tetrads, only one with three pollen, and zero with four pollen germinated in an *in vitro* assay; Among 73 sec3a heterozygous tetrads, only one with three pollen, and zero with four pollen germinated in an *in vivo* assay. Therefore, we believed that pollen germination defect, rather than pollen tube growth, is the major physiological aberrance revealed by the *sec3a/SEC3A* mutant, and our data is consistent with the report^43^. Given the fact that mutations in *SEC6, SEC8, SEC15A*, and *SEC5* dramatically affect pollen germination (1% to 7% germination rate in respective mutants) as well^22^,^23^, it is most likely that SEC3A acts together with other exocyst subunits, but as a more crucial player, in tethering secretory vesicles containing newly synthesized pectin, callose or cellulose synthase to establish and consolidate the germination aperture. It is therefore interesting to explore in the future the dynamic localization of other exocyst subunits at the germination aperture. However, other possibilities cannot be totally excluded. For example, the failure of pollen germination in *sec3a/SEC3A* mutant might be caused by defects in synthesis of some cell wall material or by an abnormal rapid degradation of cell wall materials. SEC3A appeared in a dynamic cone-shaped area and at the extreme plasma membrane at the tip of elongating pollen tube (Supplementary Video S3 and Supplementary Fig. S8) confirming recent findings^43^ and implying a role of SEC3A in pollen tube growth as well. This localization is however in contrast with an earlier report of SEC3A being localized to immobile puncta at the plasma membrane of root hairs^28^.

The expression pattern of *SEC3A* (Supplementary Fig. S1) is suggestive of a possible role of SEC3A in embryo development. Previous investigations of *sec3a* mutant line (SALK_145185) indicated that disruption of the *SEC3A* gene caused embryo lethality^28^. However, in our hand, PCR-based genotyping (Supplementary Table S1) of the SALK_145185 line did not yield any T-DNA specific band. Therefore we could not confirm the reported embryo lethality of this particular line. *PR*s*ec3a* generated in our study represented sporophytic *sec3a* homozygotes according to the genotyping results (Fig. 3), but its embryo development was normal (Supplementary Fig. S7). Our results was supported by the recent report where s*ec3a* mutant was shown to have a male transmission defect^43^.

How pollen germination site is established and pollen tube elongation initiated are not well known. In *S. cerevisiae*, bud tip localized Sec3p is thought to be a spatial landmark for polarized exocytosis and for the recruitment of other exocyst subunits to the exocytic sites^44^,^45^. The polarized localization of Sec3p is depended on its interaction with PI4,5-P_2_ and the Rho family of small GTPases^34^,^46^,^47^. In this study, SEC3A has been shown to be recruited from the cytosol to the cell cortex which marks the future germination site (Fig. 4, Supplementary Videos S1 and S2). There are two scenarios under which SEC3A could be recruited to this particular site. In plant, Rac/Rop small GTPases accumulate specifically at the plasma membrane of the tip of elongating pollen tubes and are key regulators of polar cell expansion^48^. Interestingly, Rop1 GTPase effector ICR1/RIP1, an interacting protein of SEC3A^49^, has been shown to localize and oscillate at the germination site^50^ in a similar manner to that of SEC3A (Supplementary Videos S1 and S2). Moreover, the *icr1* mutant were reported to be partially male sterile, although the details of pollen abnormality remained to be studied^49^. Under this scenario, ROP GTPase might be involved in the recruitment of exocyst to the site of pollen germination through the interaction between SEC3A and ICR1. PI4,5-P_2_ has been shown to accumulate exclusively at the apex of elongating pollen tube to control polar secretion by modulating actin organization and membrane traffic^51^. In *pip5k4* mutant where PI4,5-P_2_ level was reduced, but the position of SEC3A at the germination pore is unaffected (Fig. 4). Hence, the alternative scenario that SEC3A binds directly to PI4,5-P_2_ to achieve its polar localization is not true. Given the fact that SEC3A labeled pollen germination site (Fig. 4), mutant pollen from *sec3a/SEC3A* plant could not germinate, and partial complementation of *sec3a* resulted in multiple germination sites^43^, we suggest that SEC3A is required for germination site selection and/or establishment.

## Materials and Methods

### Plant materials and growth conditions

*Arabidopsis thaliana* Columbia ecotype (Col-0) was used as the wild type. T-DNA insertion lines of *SEC3A* (GK_652H12) and *PIP5K4* (SALK_001138) were obtained from the Nottingham Arabidopsis Stock Center (NASC) and Arabidopsis Biological Resource Center (ABRC), respectively. Seeds were surface sterilized and plated on half-strength Murashige and Skoog medium with 0.8% agar, imbibed at 4 °C for 3 days, and then placed in a growth chamber at 22 °C with a 16-h-light/8-h-dark cycle. Seven-day-old seedlings were then transferred to soil and maintained under the same condition.

### GUS staining

For GUS staining, different tissues of transgenic Arabidopsis plants were vacuum infiltrated for 15 min in GUS staining solution of 100 mM phosphate buffer (pH 7.0), 0.1% Triton X-100, 0.5 mM [K_3_Fe(CN)_6_], 0.5 mM [K_4_Fe(CN)_6_], 10 mM EDTA and 0.5 mg ml^−1^ bromochloroindoyl-β-glucuronide (X-Gluc). Samples were then incubated overnight at 37 °C and cleared in acetic acid:ethanol (1:3 v/v).

### Phenotypic analysis of mutants

To determine different pollen development stages, pollen grains were stained in a DAPI solution (0.1 M phosphate buffer solution pH 7.0, 1 mM EDTA, 0.1% Triton X-100 and 1 μg ml^−1^ DAPI) for 15 min before observation. The viability of pollen grains was assessed using Alexander staining^52^. For SEM, mature pollen grains were coated directly with gold particles (EIKO IB-3) and observed on HITACHI S-3000N scanning electron microscope.

*In vitro* pollen germination was conducted essentially according to described previously^53^. Pollen harvested from newly fully opened flowers was placed onto pollen germination medium (PGM) consisting of 1 mM CaCl_2_, 1 mM Ca(NO_3_)_2_, 1 mM MgSO_4_, 0.01% (w/v) H_3_BO_3_, 18% (w/v) sucrose, pH 7.0, which was solidified with 0.8% (w/v) agar, and grown in a growth chamber in the dark at 22 °C.

For *in vivo* pollen germination assay, mature pistils of the male-sterile mutant *ms1*^32^ were pollinated with a limited number (3–5) of quartets from *sec3a/SEC3A qrt1/qrt1* and *qrt1/qrt1* plants. The pollen tubes in the pistils were stained with aniline blue and viewed with a confocal microscope Zeiss LSM710 system.

### Complementation experiments

For the pollen-rescue experiment, the *LAT52* promoter and the coding sequence (CDS) of *SEC3A* were amplified by PCR using the primer pairs *LAT52-S (SacI)/LAT52-A (KpnI)* and *SEC3A1-S (BamHI)/SEC3A1-A (NheI)*, respectively (Supplementary Table S1). The resulting DNA fragments were cloned into the pCAMBIA1300 vector (CAMBIA, http://www.cambia.org) and sequencing validated. For genomic DNA complementation experiment, about 1.1 kb promoter region along with 8230 bp genomic sequence of *SEC3A* was PCR-amplified using the primer pair *SEC3A2-S/SEC3A2-A* (Supplementary Table S1). The amplified fragment was cloned into the vector pCAMBIA1300221 (CAMBIA, http://www.cambia.org) using in-fusion HD cloning kit according to the manufacturer’s instructions (Clontech). The above constructs were introduced into the *sec3a/SEC3A* plants using the Agrobacterium (strain EHA105)-mediated infiltration method^54^, followed by hygromycin selection.

### Confocal microscopy

Confocal images of pollen grains or pollen tubes were collected by UltraView spinning-disc confocal scanner unit (Perkin Elmer). The wavelength was 488 nm for GFP excitation and 505–530 nm for detection. The excitation and detection wavelengths for DAPI and Calcofluor white were 359 nm and 385–405 nm for excitation, 461 nm and 437–445 nm for detection,

### PIP strip overlay binding assay

For the PIP strip binding assays, phospholipid membranes (Echelon) were blocked with 3% bovine serum albumin (BSA) in PBS-T (0.1% v/v Tween-20) for 1 h at room temperature. After that, membranes were incubated in a buffer with or without SEC3A-HIS fusion proteins (0.5 μg ml^−1^) in 3% BSA/PBS-T for another hour. Unbound protein was washed away with PBST for three times, 10 min each. The membranes were then incubated with anti-HIS antibodies to detect the bound proteins.

### Immunofluorescence and cytochemical staining

Pollen grains were adhered to poly-L-lysine-covered glass slides after germination in liquid PGM for 30 min. Samples were fixed in 4% (w/v) polyformaldehyde in PIPES buffer (50 mM PIPES, 1 mM EGTA, 5 mM MgSO_4_, 0.5 mM CaCl_2_, 0.1% TritonX-100, pH 7) for 1 h. After washing with PBS (100 mM potassium phosphate, 138 mM NaCl, and 2.7 mM KCl, pH 7.3), samples were incubated in the blocking buffer (0.8% BSA, 0.1% gelatin, and 2 mM NaN_3_ in PBS) at room temperature for 30 min and then incubated with the primary antibodies (1:200 diluted in blocking buffer) for 1 h. Pectins with low and high degrees of methylesterification were labeled with JIM5 and JIM7^55^,^56^, respectively (Plant Probes). Callose was labeled with anti-callose^57^ (Biosupplies Australia Pty Ltd.). The pollen grains were washed three times with PBS and incubated for 30 min with Alexa Fluor 594 conjugated secondary antibodies (1:100 dilution in blocking buffer). The samples were washed for five times with PBS before analysis using an UltraView spinning-disc confocal scanner unit (Perkin Elmer). For cytochemical staining, the pollen tubes were stained without fixation after germination in liquid PGM. Calcofluor white (0.001%, w/v) and ruthenium red (0.01%, w/v) in PGM were used to stain β-glucans (both cellulose and callose) and pectins, respectively. Chemicals used are from Sigma-Aldrich.

## Additional Information

**How to cite this article**: Li, Y. *et al*. Exocyst subunit SEC3A marks the germination site and is essential for pollen germination in *Arabidopsis thaliana. Sci. Rep.* **7**, 40279; doi: 10.1038/srep40279 (2017).

**Publisher's note:** Springer Nature remains neutral with regard to jurisdictional claims in published maps and institutional affiliations.

## Supplementary Material

Supplementary Figure

Supplementary Video. S1

Supplementary Video. S2

Supplementary Video. S3

## Figures and Tables

**Table 1:** Genotyping of the *sec3a/SEC3A* mutant.

	No. of Progeny	Genotypes of Progeny			χ^2^	*P*
		*SEC3A/SEC3A*	*sec3a/SEC3A*	*sec3a/sec3a*		
Self-cross		25 (%)	50 (%)	25 (%)	Expected	
	243	48	52	0	125.7	<0.001^a^
Out-cross (♀ × ♂)		50 (%)	50 (%)	0 (%)	Expected	
WT × *sec3a/SEC3A*	224	100	0	0	224	<0.001^a^
*sec3a/SEC3A* × WT	271	51	49	0	0.1328	NS

*P*, values were calculated using the χ^2^ test.

^a^Significant difference between the observed and the expected ratios.

NS, Not significantly different.

**Table 2:** Complementation analysis of *sec3a/SEC3A* mutants.

Complemented T_1_	No. of Progeny	Genotypes of Progeny			χ^2^	*P*
		*SEC3A/SEC3A*	*sec3a/SEC3A*	*sec3a/sec3a*		
		33.3 (%)	50 (%)	16.7 (%)	Expected	
*pLAT52:SEC3A* 1^#^	249	35.3	42.1	22.6	8.42	0.0148
*pLAT52:SEC3A* 2^#^	194	29.4	54.1	16.5	1.56	NS
*pLAT52:SEC3A* 3^#^	201	27.9	48.8	23.3	7.31	0.0258
*pLAT52:SEC3A-GFP* 1^#^	204	37.7	50.5	11.8	2.34	NS
*pLAT52:SEC3A-GFP* 2^#^	166	32.5	52.4	15	0.08	NS

*pLAT52:SEC3A* represents *pLAT52:SEC3A* hemizygous transgene in *sec3a/SEC3A* mutant background.

*pLAT52:SEC3A-GFP* represents *pLAT52:SEC3A-GFP* hemizygous transgene in *sec3a/SEC3A* mutant background.

*P*, values were calculated using the χ^2^ test.

NS NS, Not significantly different.
