# Supplementary material for: Exocyst subunit SEC3A marks the germination site and is essential for pollen germination in Arabidopsis thaliana
Source: Sci Rep. 2017 Jan 11;7:40279. doi: 10.1038/srep40279 (PMC5225640; doi:10.1038/srep40279)
Supplement: Supplementary Figure [file srep40279-s1.pdf]

Exocyst subunit SEC3A marks the germination site and is essential for pollen germination in *Arabidopsis thaliana*

Yan Li, Xiaoyun Tan, Mengru Wang, Bingxuan Li, Yanxue Zhao, Chengyun Wu, Qingchen Rui, Junxia Wang, Zhongyuan Liu and Yiqun Bao[1]

College of Life Sciences, Nanjing Agricultural University, Nanjing 210095, People's Republic of China

[1]To whom correspondence should be addressed: E-mail: baoyiqun@njau.edu.cn

Fax: +86-25-84396795

Supplementary Video. S1 online: SEC3A proteins marks the pollen germination site during pollen germination. Bars= 15 μm.

Supplementary Video. S2 online: The localization of SEC3A in the growing pollen tube. Bars= 20 μm.

Supplementary Video. S3 online: The localization of SEC3A during pollen tube growth. Bars= 20 μm.

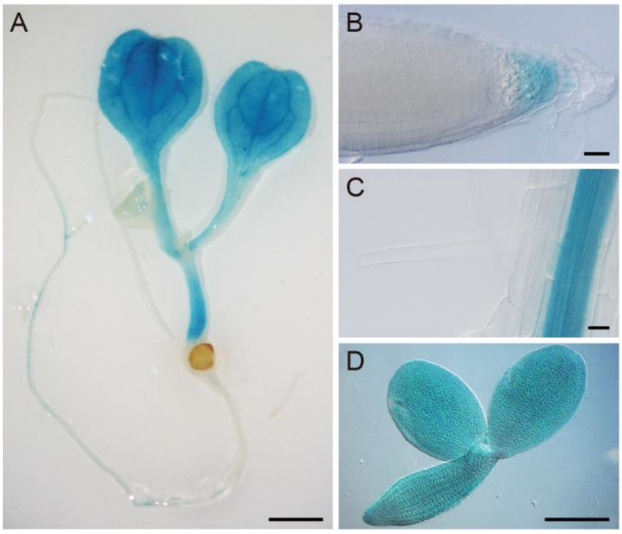

Supplementary Figure S1 online: Expression of *SEC3A* was detected in the seedling (**A**), the root columella cells (**B**), the vascular bundles of root maturation zone (**C**), and the embryo (**D**). Bars= 1 mm for (**A**), 20 μm for (**B**, **C**), and 200 μm for (**D**).

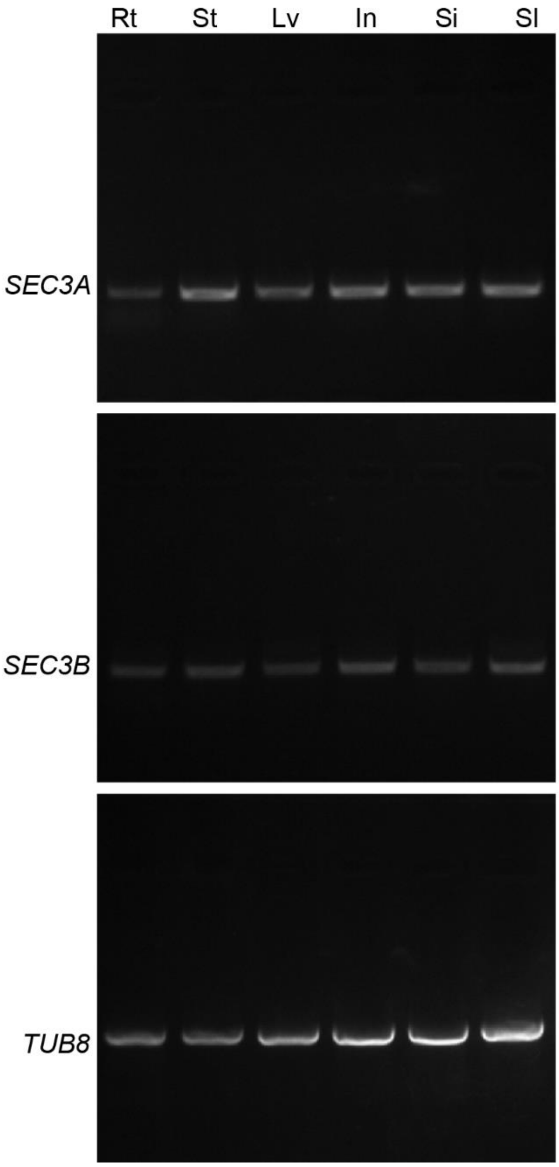

Supplementary Figure S2 online: RT-PCR analysis of *SEC3A* and *SEC3B* gene expression. Rt, root; St, stem; Lv, mature leaves; In, inflorescence; Si, siliques; Sl, seedlings. *TUB8* was used as an internal loading control.

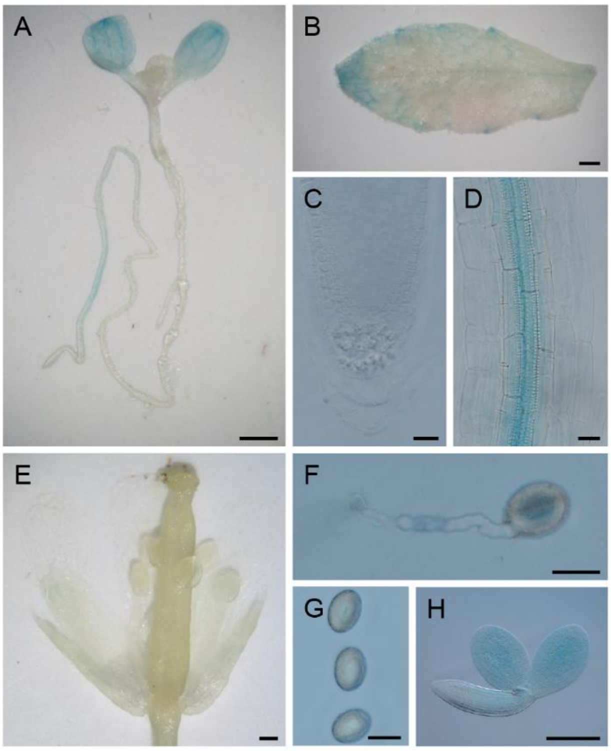

Supplementary Figure S3 online: Expression pattern of *SEC3B* in Arabidopsis. (**A**) Seedlings, (**B**) mature leaves, (**C**) root tip, (**D**) root vasculature, (**E**) flower, (**F**) pollen tube, (**G**) pollen grain and (**H**) embryo. Bars= 1 mm for (**A**, **B**), 20 μm for (**C**, **D**, **F**, **G**) and 200 μm for (**E**, **H**).

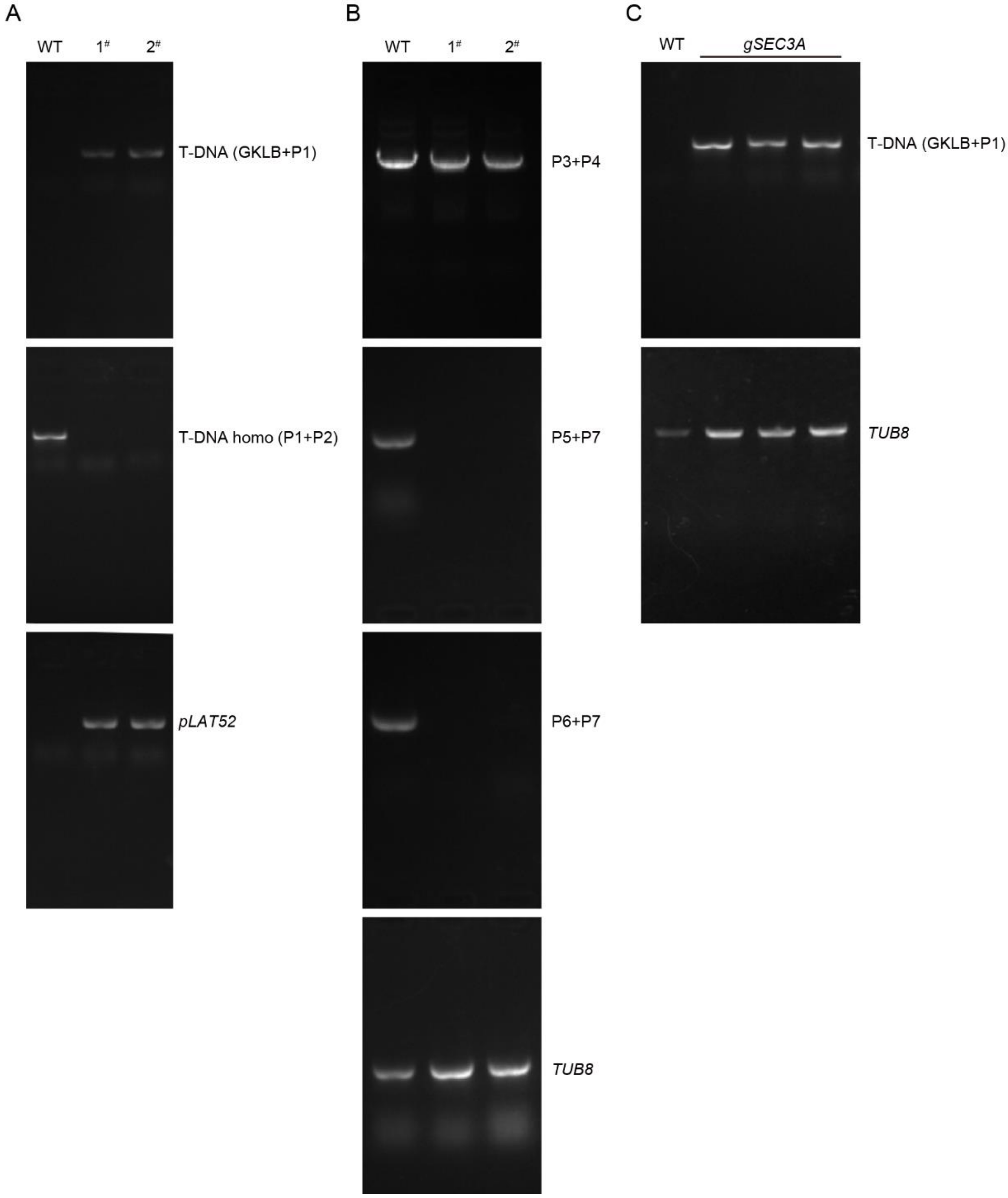

Supplementary Figure S4 online: Complementation analysis of *sec3a* mutants with *pLAT52:SEC3A-GFP* or *gSEC3A* transgene. (**A**) Genotyping of *PRsec3a-GFP* 1# and 2# lines by PCR analysis. (**B**) The expression of different *SEC3a* transcripts in *PRsec3a-GFP* 1# and 2# lines. (**C**) Genotyping of *sec3a/SEC3A* mutant plant bearing *gSEC3A* hemizygous transgene. *TUB8* was used as an internal control.

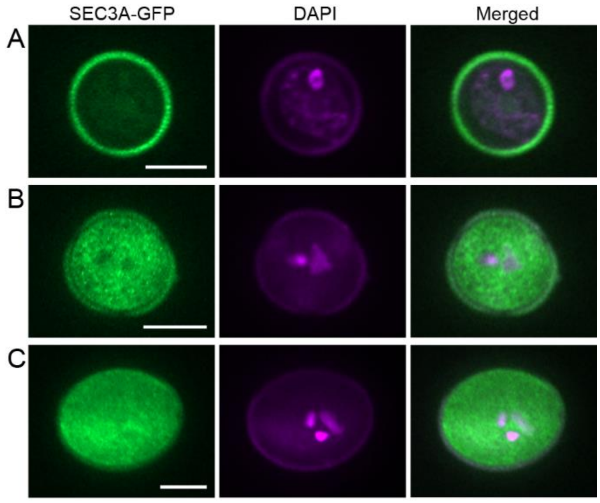

Supplementary Figure S5 online: SEC3A proteins expressed from the bicellular stage onwards. (**A**-**C**) Confocal images of SEC3A (green) and DAPI (magenta) signals in pollen at stages of Microspore (**A**), Bicellular (**B**), and Tricellular (**C**). Bars= 10 μm.

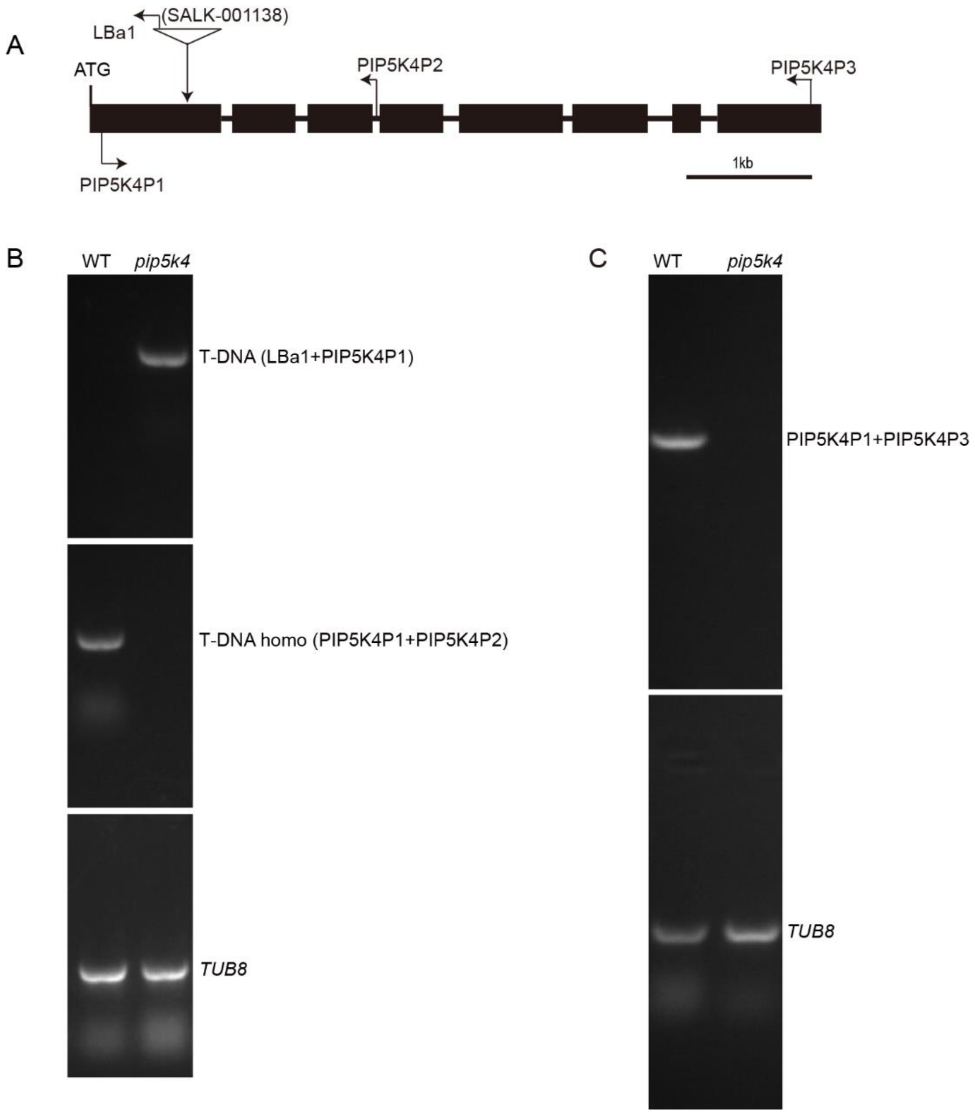

Supplementary Figure S6 online: Genotyping of *pip5k4* mutant (SALK_001138).

(**A**) Graphical representation of the position of insertion. (**B**) Genotyping results showed that this mutant is a homozygote. (**C**) RT-PCR analysis showed the absence of *PIP5K4* transcripts in homozygous *pip5k4* line. *TUB8* was used as an internal control.

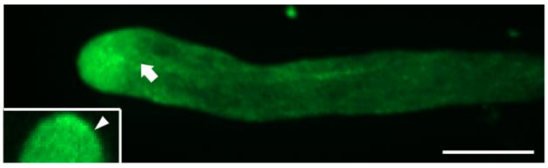

Supplementary Figure S7 online: The localization of SEC3A in pollen tube.

Arrows and arrowhead indicated the prime localization of SEC3A. Bars=10 μm.

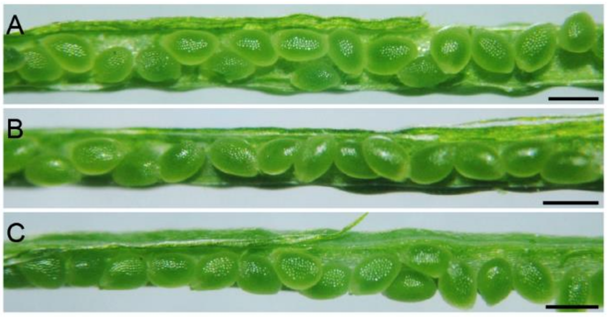

Supplementary Figure S8 online: The *PRsec3a* embryos are normal. Wild type (**A**), *PRsec3a* 1# (**B**) and *PRsec3a* 2# (**C**) developing seeds within the siliques. Bars= 0.5 mm.

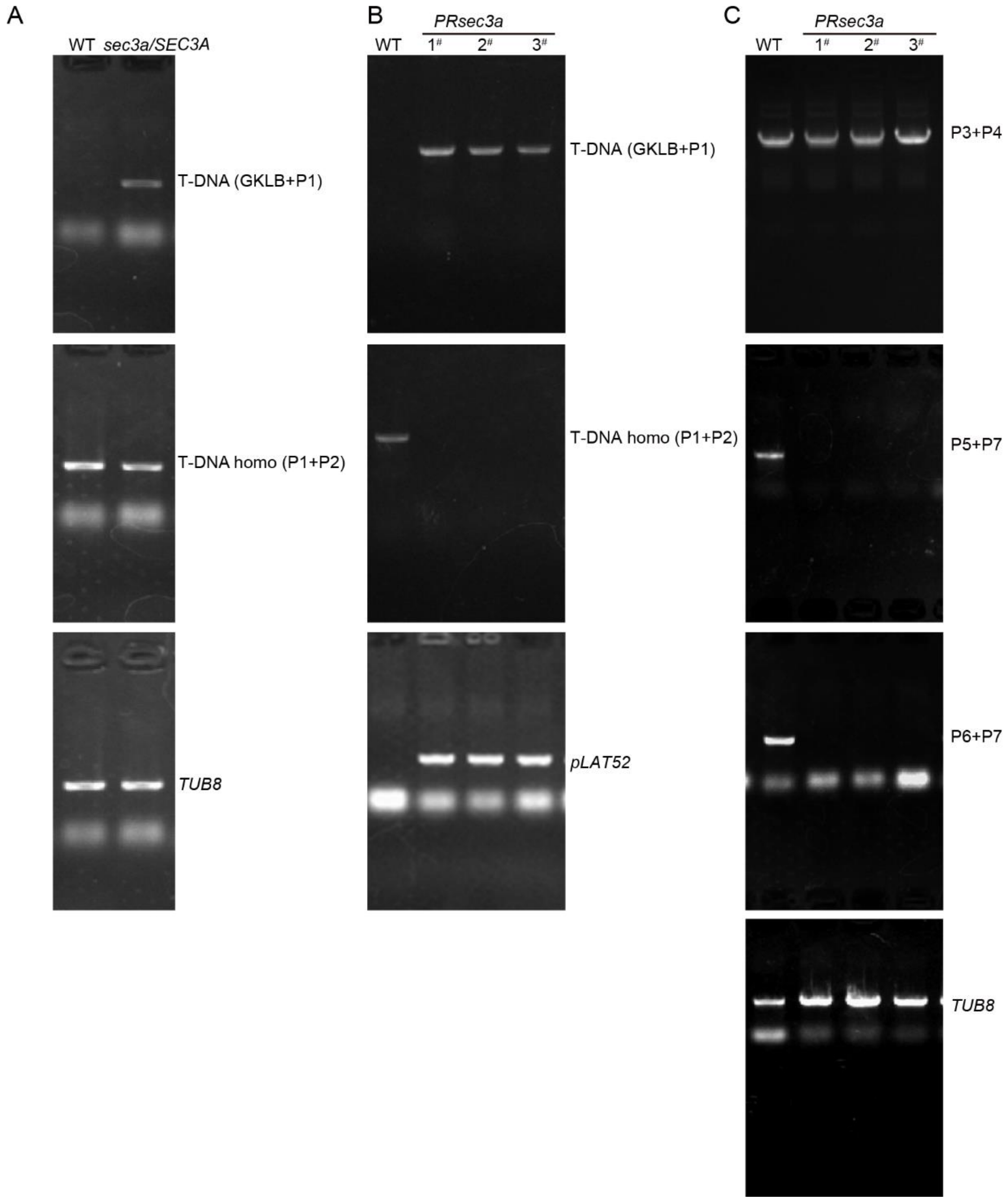

Supplementary Figure S9 online: (A) Full-length gel of Figure 1H. **(B)** Full-length gel of Figure 3A. **(C)** Full-length gel of Figure 3B.

Usage	Primer Name	Oligo nucleotide（5’-3’）
**Genotyping and transgenic plant identification**	GK-LB	ATATTGACCATCATACTCATTGC
	LBa1	TGGTTCACGTAGTGGGCCATCG
	P1	CTTCCCAATTGTTCATACTTCC
	P2	CTCCAGGAACGTTTTCACTC
	P3	ATGGCGAAATCAAGCGCCGA
	P4	GTAGCAACTCCTCTGATGCTAG
	P5	GTGGACAAATCGATTGCAGCA
	P6	ACGAGAGCTTCGTGCAGCTC
	P7	CGACTCTGCTAAGAGAGCCA
	P8	TTTCGTGGACCCTTTGATTT
	PIP5K4P1	TGAACGGTACTTATTATCCATCAGG
	PIP5K4P2	AAGCACCTGCATTATATAATTCCAG
	PIP5K4P3	ACGTCTCGAGTACAGCCTCG
	LAT52P1	GCATGCCTGCAGGTCGAC
	LAT52P2	GATCCTCTAGACTCCATGG’
	TUB8-S	CTTCGTATTTGGTCAATCCGGTGC
	TUB8-A	GAACATGGCTGAGGCTGTCAAGTA
	SEC3A RT-S	ATGGCCAGTTTCCTCAGTG
	SEC3A RT-A	AGCCATCCAAATCGAGAGC
	SEC3B RT-S	GTGATTGAACCAAAACTGAAGGC
	SEC3B RT-A	ACATCACTTGTATCCGTGA
**Gene expression and protein localization constructs**	PSEC3A:GUS-S	ggaattcGGCCAACTTCTTTGTCTCTGTCTTT
	PSEC3A:GUS-A	aactgcagTGTTGTTGTTGCGGATCCAGC
	PSEC3B:GUS-S	ggaattcGAAGACGAGAGTTCAGATTGGTCGC
	PSEC3B:GUS-A	aactgcagTGTTGTTGTTGCGGATCCAGC
	pLAT52 -S	cgagctcATACTCGACTCAGAAGGTATTG
	pLAT52 -A	ggggtaccTAATTGGAAATTTTTTTTTTGG
	SEC3A1-S	cgggatccATGGCGAAATCAAGCGCCGA
	SEC3A1-A	ctagctagcCATGGAAGCCAGAAGTCCTCTC
	SEC3A2-S	agcttgcatgcctgcagGGCCAACTTCTTTGTCTCTGTC
	SEC3A2-A	cggggaaattcgagctcGAGTGGTCTTCTGCCTACATAAG

Supplementary Table S1 online: List of primer pairs used in this study.

Complemented T_1	No. of Progeny	With insertion	Without insertion	Ratio
gSEC3A 1#	207	132	75	1.76
gSEC3A 2#	235	145	90	1.61

gSEC3A represents *pSEC3A:gSEC3A* hemizygous transgenic line in *sec3a/SEC3A* background.

Ratio = With insertion/Without T-DNA insertion.

Supplementary Table S2 online: Complementation analysis of *sec3a/SEC3A* mutants.
